# Integration of transcriptomic data identifies key hallmark genes in hypertrophic cardiomyopathy

**DOI:** 10.1186/s12872-021-02147-7

**Published:** 2021-07-06

**Authors:** Jing Xu, Xiangdong Liu, Qiming Dai

**Affiliations:** 1 Department of Clinical Laboratory, ZhongDa Hospital, Southeast University, Nanjing, China; 2 Institute of Life Science, Southeast University, Nanjing, China; 3 Department of Cardiology, ZhongDa Hospital, Southeast University, Nanjing, China

**Keywords:** Hypertrophic cardiomyopathy, Microarray, RNA-Seq, Classification, JAK2

## Abstract

**Background:**

Hypertrophic cardiomyopathy (HCM) represents one of the most common inherited heart diseases. To identify key molecules involved in the development of HCM, gene expression patterns of the heart tissue samples in HCM patients from multiple microarray and RNA-seq platforms were investigated.

**Methods:**

The significant genes were obtained through the intersection of two gene sets, corresponding to the identified differentially expressed genes (DEGs) within the microarray data and within the RNA-Seq data. Those genes were further ranked using minimum-Redundancy Maximum-Relevance feature selection algorithm. Moreover, the genes were assessed by three different machine learning methods for classification, including support vector machines, random forest and k-Nearest Neighbor.

**Results:**

Outstanding results were achieved by taking exclusively the top eight genes of the ranking into consideration. Since the eight genes were identified as candidate HCM hallmark genes, the interactions between them and known HCM disease genes were explored through the protein–protein interaction (PPI) network. Most candidate HCM hallmark genes were found to have direct or indirect interactions with known HCM diseases genes in the PPI network, particularly the hub genes *JAK2* and *GADD45A*.

**Conclusions:**

This study highlights the transcriptomic data integration, in combination with machine learning methods, in providing insight into the key hallmark genes in the genetic etiology of HCM.

**Supplementary Information:**

The online version contains supplementary material available at 10.1186/s12872-021-02147-7.

## Background

Hypertrophic cardiomyopathy (HCM) is a genetically heterogeneous cardiac muscle disorder characterized by left ventricle hypertrophy in the absence of abnormal loading conditions [1]. HCM occurs in at least 1 in 500 of the general population, making it one of the most common inherited heart diseases [2]. In 70% of HCM patients, the disease is caused by mutations in sarcomeric genes, Z-disc genes, calcium-handling genes and so on. The genetic background of about 30% HCM patients remains unknown [3]. The cellular signaling processes that lead from the primary mutation to the HCM phenotype are also poorly understood. Therefore, it is essential to investigate the pathogenic mechanisms and develop novel diagnostic hallmark genes.

Two gene expression profiling technologies, microarray and RNA sequencing (RNA-Seq), have been widely used for obtaining gene expression signature. Compared to microarray, RNA-Seq can simultaneously detect whole gene expression levels [4]. Existing evidence showed a high consistency between microarray and RNA-Seq [5, 6].

In the last decade, various methods for classification have been developed and gained great attention of biomedical applications [7, 8]. In most classification studies, support vector machines (SVM), random forest (RF), K-Nearest-Neighbors (KNN) are reported as the foremost classifiers producing high accuracies [9].

**Fig. 1:**
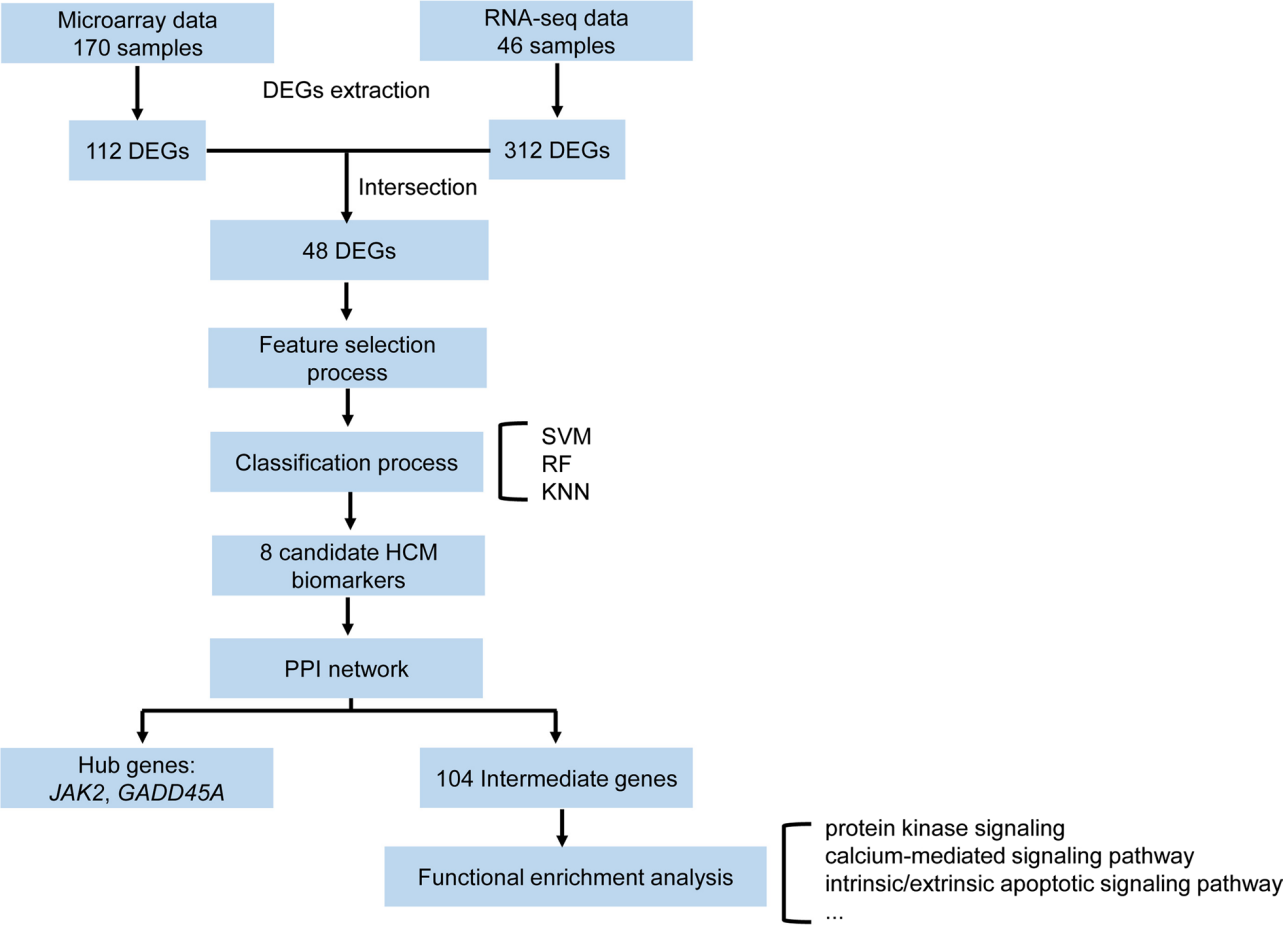
Flowchart of using transcriptomic data for HCM hallmark genes discovery

In this study, the integrated analysis of transcriptomic datasets from different platforms was performed to identify differentially expressed genes (DEGs) between HCM patients and healthy controls (Fig. 1). Machine learning methods, including SVM, RF and KNN, were applied to prioritize the HCM candidate hallmark genes. This study provided novel perspective for understanding mechanism and exploiting new therapeutic means for HCM.

## Methods

### Data collection

Gene expression profiles of the heart tissue samples in HCM patients/mice and healthy controls and patient-specific induced pluripotent stem cells-derived cardiomyocytes (iPSC-CMs) were obtained from ArrayExpress (http://www.ebi.ac.uk/arrayexpress/), Gene Expression Omnibus (GEO, https://www.ncbi.nlm.nih.gov/gds/) and Sequence Read Archive (SRA, https://www.ncbi.nlm.nih.gov/sra/).

### Microarray data analysis

For microarray datasets, standard analysis process including quality control, pre-processing, normalization using *Limma* and *Lumi* packages across Illumina and CapitalBio platforms was performed [10, 11]. To avoid distortion of the results by noise, uninformative probes (low variance, expressed uniformly close to background detection levels) were filtered out. Finally, normalized log2-transformed expression values were obtained.

### RNA-seq data analysis

For RNA-Seq data sets, after removing adapters and low-quality bases using the Trimmomatic program, we implemented *STAR* [12] to map reads to human genome hg38. *Samtools* [13] and *Htseq* [14] were then used to obtain the read count for each gene. Then the expression values for the genes were calculated using the *Cqn* and the *NOISeq* R packages [15, 16].

### DEGs extraction

The expression values obtained from both microarray and RNA-Seq technologies were integrated using the *merge* function from the base R package. Extraction of DEGs was performed using the *limma* R package, at both individual level (microarray data and RNA-Seq data separately). Then a normalization of all joint data was applied using the *NormalizedBetweenArrays* function. Log-fold change (LFC) and adjusted p value (adj. PV) using Benjamini Hochberg's method, were considered to select statistically highly differentiated expressed genes.

### Feature selection process

The feature selection process was performed to obtain a ranking of the most relevant DEGs, using the minimum-Redundancy Maximum-Relevance (mRMR) algorithm [17]. To create this ranking, mRMR sorts the genes so that they bring largest relevance with respect to the class (HCM/control), at the same time, they have lowest redundancy among themselves. Therefore, this algorithm will rank in first position the gene that contains the largest amount of information, but the following genes will provide also minimum redundancy (apart from maximum relevance as regard to the class) with respect to the already selected genes. The mRMR algorithm was implemented by importing the *pymrmr* package with python [18].

### Classification process

In the classification process, three different machine learning algorithms, including SVM, RF and KNN, were implemented to assess the results. The experiments are implemented with Python using the *svm*, *RandomForestClassifier*, *KNeighborsClassifier* from scikit-learn libraries [19].

SVM are supervised learning models with associated learning algorithms that analyze data used for classification and regression analysis [20]. Four kernels, including the *radial basis function* (RBF), *polynomial*, *linear* and *sigmoid* kernel, were tested to implement the SVM algorithm. Among the four kernels, the RBF kernel showed a good performance and was chosen by using the argument (kernel = 'rbf').

RF is essentially, an ensemble of decision trees combined where each tree votes on the class assigned to a given sample, with the most frequent answer winning the vote [21]. For the RF classifier, two main parameters were tested and evaluated: n_estimators and min_samples_leaf.

The KNN algorithm is an instance-based learning method for classifying objects based on closest training examples in the feature space [22]. Two main parameters n_neighbors and p were tested to find the optimal KNN model for classification.

Ten-fold cross-validation (CV) was used over the training dataset to obtain the optimal hyperparameters for the methodologies. Accuracy and f1-score were used as the performance measures.

### Protein interaction network

The protein–protein interaction (PPI) network is represented as graphs where nodes and edges are proteins and pair wise interactions, respectively. Only intermediate genes known to interact between 47 known HCM disease genes and HCM candidate hallmark genes were included. Experimentally verified interaction data from StringDB [23] and Biogrid [24] were used for establishing the PPI network. Only medium- and high-confidence experimental interactions in StringDB were shown, although these may not always represent local interactions. Cytoscape (version 3.7.2), a bioinformatics software platform, was used for visualizing the molecular interaction networks [25]. ClueGO, a cytoscape plugin, was used for functional enrichment analysis based on the intermediate genes [26].

### Statistical analysis

Statistical analysis and Pearson’s correlation analysis were performed with R studio.

## Results

### Integration of samples

**Fig. 2:**
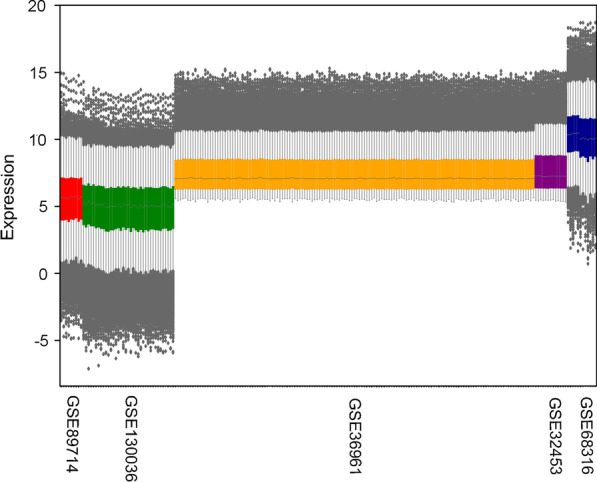
Expression profile of each dataset before normalization

**Fig. 3:**
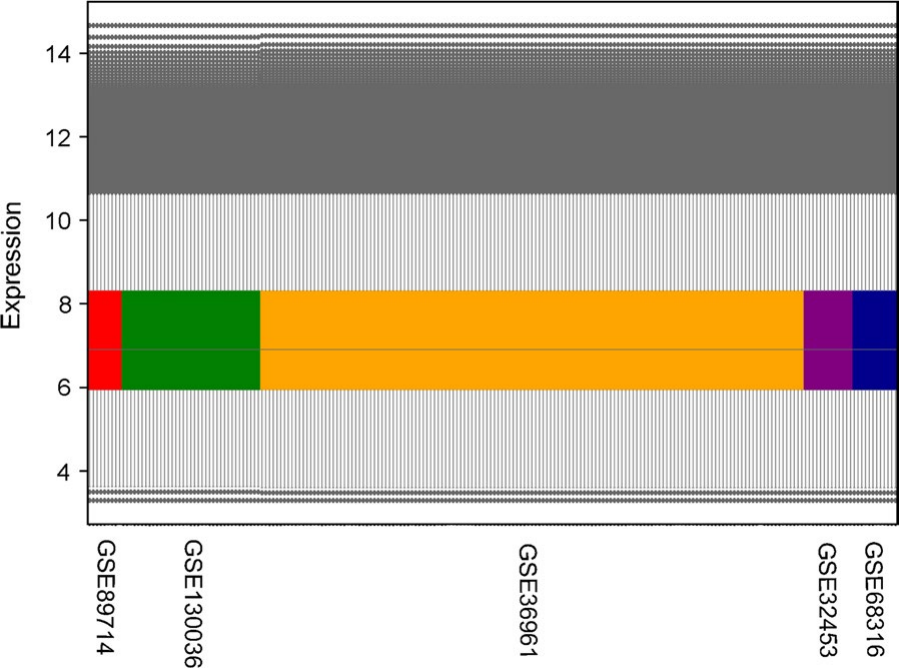
Expression profile of each dataset after normalization

Two hundred sixteen samples in 5 datasets were selected, including 154 HCM samples and 62 healthy control samples (Table 1). Four datasets contain gender information, and one of them with *MYH7*/*MYBPC3* genotype information. Both microarray and RNA-Seq data analysis were conducted and the gene expression values were obtained for each technology separately. The representation of the individual dataset reflected several different expression value ranges (Fig. 2). To remove dynamic expression variability between samples due to different platforms, a normalization of all joint data per technology was performed (Fig. 3).

**Table 1:** Characteristics of microarray and RNA-Seq datasets in the study

Datasets	Source	Type	Technology	Platform	Year	Case/Control
E-GEOD-36961	Human heart tissue	mRNA	Microarray	Illumina GPL15389	2012	106/39
GSE32453	Human heart tissue	mRNA	Microarray	Illumina GPL6104	2012	8/5
E-GEOD-68316	Human heart tissue	mRNA	Microarray	CapitalBio GPL20113	2015	7/5
*Integrated (Microarray)*						121/49
GSE89714	Human heart tissue	mRNA	RNA-Seq	Illumina GPL20795	2016	5/4
GSE130036	Human heart tissue	mRNA	RNA-Seq	Illumina GPL11154	2019	28/9
Integrated (RNA-Seq)						33/13

*Source*: GEO/ArrayExpress accession

### Detection of DEGs

**Fig. 4:**
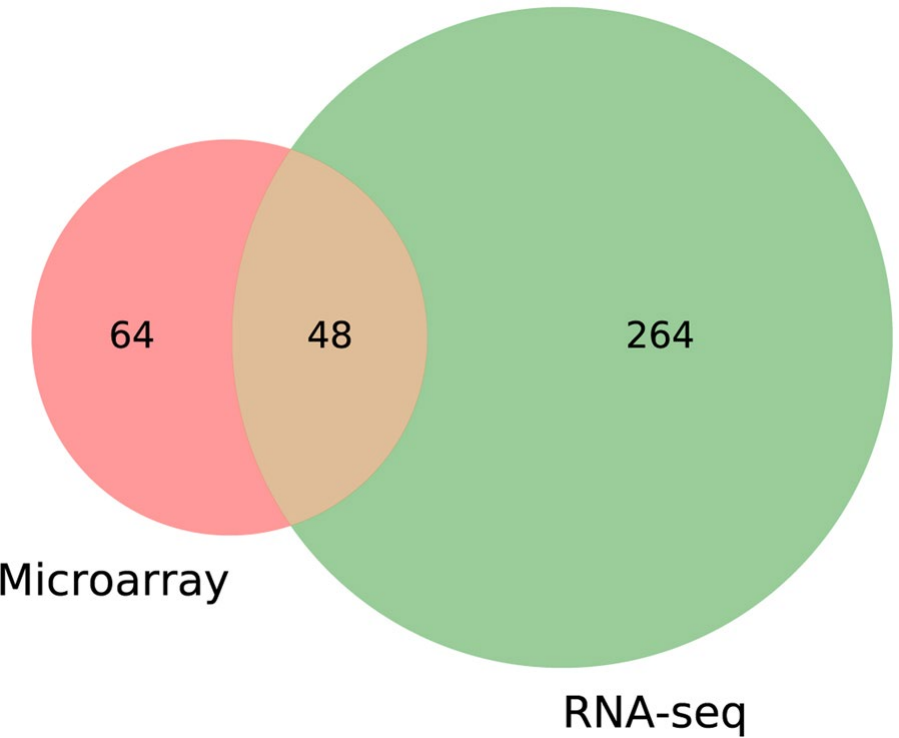
Intersection of DEGs in RNA-Seq dataset and microarray dataset

After data integration, the general LFC value of the identified DEGs were relatively low with a maximum value of 2.36. Therefore, the criteria for DEGs detection we chosen was less stringent, with |LFC|≥ 0.6, and |adj. PV|≤ 0.05. Two sets of DEGs were identified for microarray dataset and RNA-Seq dataset (Fig. 1). A total of 48 common DEGs were obtained through the intersection of the two sets of DEGs (Fig. 4). Two genes (*GADD45B* and *THBS1*) showed opposite direction in the two DEGs sets (Additional file 1: Table S1 and S2).

### Assessment of DEGs

The feature selection process was applied to the 48 DEGs, and the ranking of the genes was based on its relevance with HCM using the mRMR algorithm. Subsequently, the performance of the obtained ranking was evaluated. Stratified sampling was used to divide the integrated dataset into a training dataset (172 samples) and a test dataset (44 samples). The expression values of the 48 DEGs were chosen as classification features. Three different classifiers were implemented and compared, including SVM [20], RF [21] and KNN [22]. Furthermore, the comparison has been performed for both accuracy and f1-score with different number of genes. The f1-score is a measure of a test's accuracy, calculated by using both the precision or accuracy and the recall or sensitivity.

The validation results (10-CV over the training dataset) and test results using the three classifiers were shown in Additional file 1: Table S3. These validation results were above 87% using only the first gene of the ranking for classification, and above 93% using a reduced set of eight genes in the ranking. Using those eight genes, the test results showed an accuracy of 97.73% using SVMs, 100% using KNN, nevertheless lower using RF with a 93.18%. Consequently, the main set of 48 DEGs was reduced to the eight genes, which allow discerning whether new samples are HCM or not. The eight genes (*JAK2*, *C1R*, *MS4A7*, *MBP*, *METTL7B*, *GADD45A*, *CD209*, *TRAK2*) were then listed as candidate HCM hallmark genes.

**Fig. 5:**
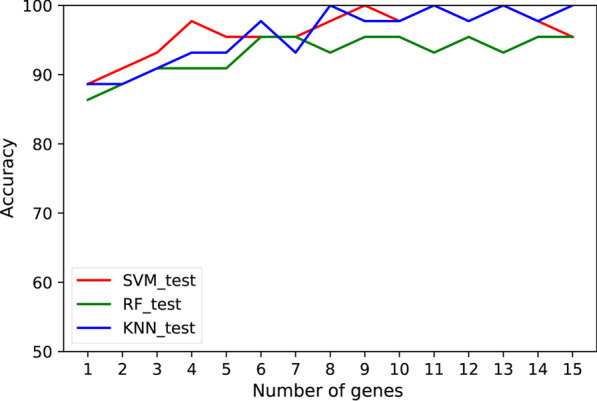
The test accuracy achieved by SVM, RF and KNN using the most relevant genes obtained by mRMR. Similar trends can be observed for f1-score

**Fig. 6:**
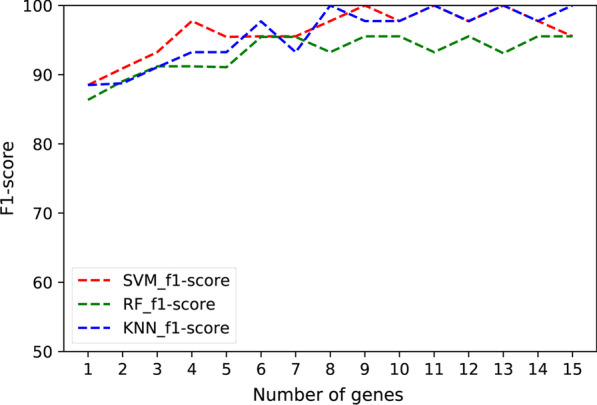
The f1-score achieved by SVM, RF and KNN using the most relevant genes obtained by mRMR

**Fig. 7:**
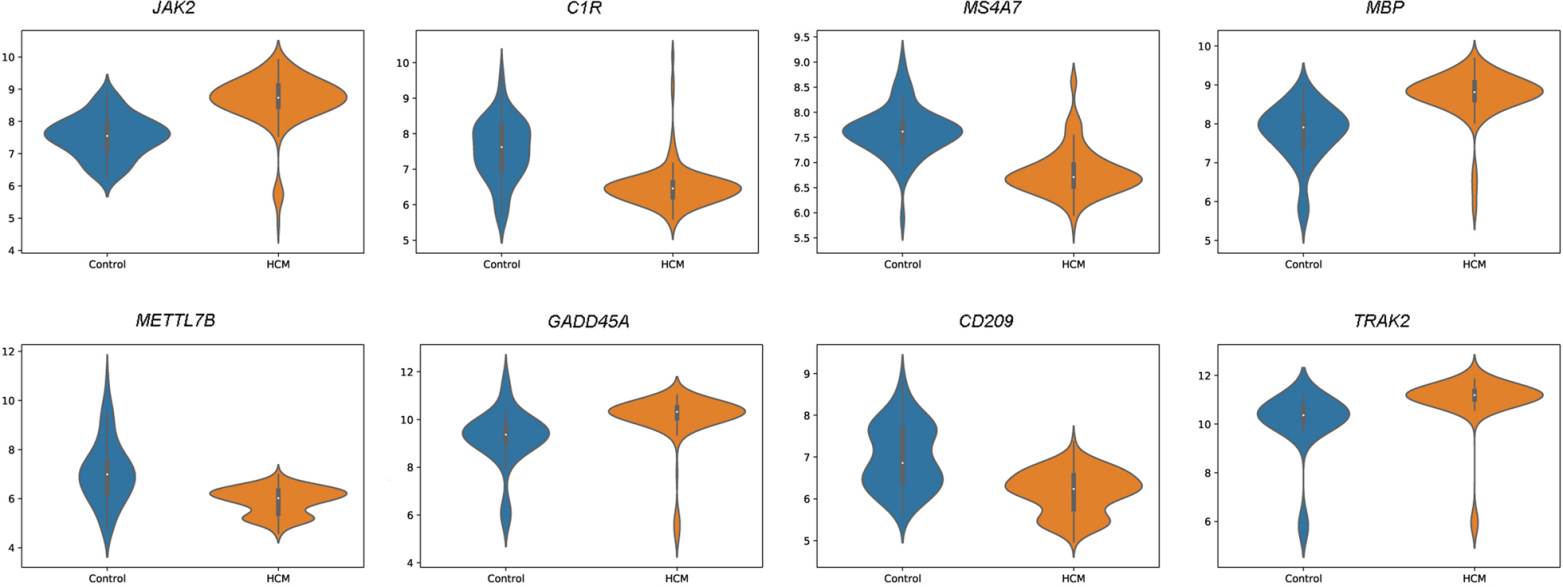
Average expression value violin plots of the eight candidate HCM hallmark genes obtained in this study

Figures 5 and 6 showed the evolution of accuracy and f1-score for the three classifiers using a different number of genes. Regarding the three classifiers, SVM reached comparable results with KNN, better than RF. Expression levels of the eight candidate HCM hallmark genes were shown in Fig. 7, revealing a clear differentiation between the average value of the HCM and healthy control samples.

To see whether gender and *MYH7*/*MYBPC3* genotype affects the expression of the eight candidate HCM hallmark genes, comparisons of the expression values were performed using student's t-test. The results showed a significant difference in the expression of the eight genes between male HCM hearts and male control hearts, as well as between female HCM hearts and female control hearts, while no significant difference was noted between male and female HCM samples. Moreover, no significant difference in the expression of the eight genes was noted between *MYH7*/*MYBPC3* genotype positive and negative HCM samples.

The expression of the 48 HCM relevant genes was also explored in the iPSC-CMs from a family cohort carrying a hereditary HCM missense mutation (Arg663His) in the *MYH7* gene (GSE35229). The expression of one candidate hallmark gene *METTL7B* was significantly increased in iPSC-CMs compared with human embryonic stem cells (hESCs) and fibroblasts (p < 0.01) [27]**.**

### Protein interaction network

**Fig. 8:**
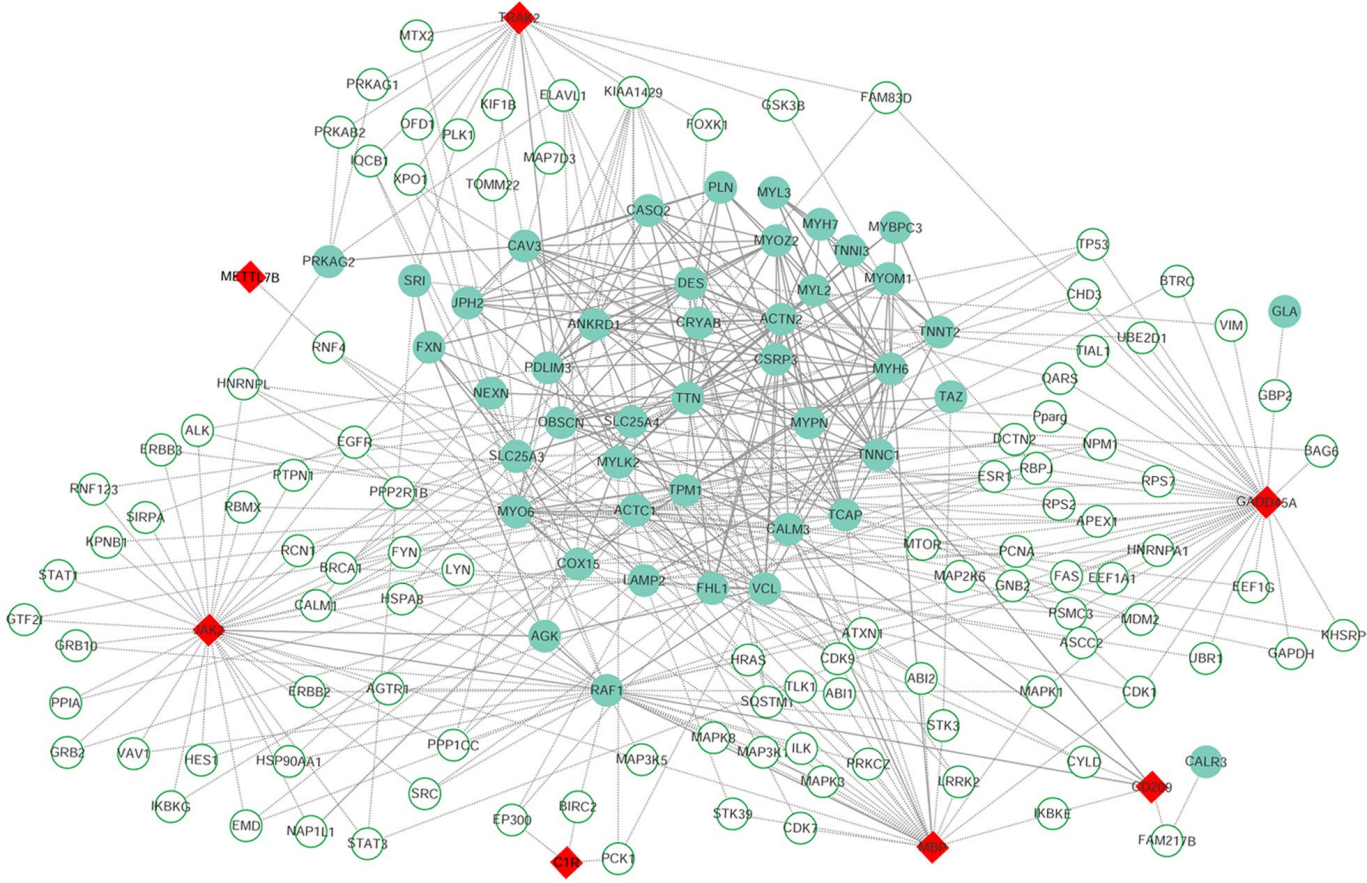
Protein–protein interaction network created by known HCM disease genes (green circle) and candidate HCM hallmark genes (red circle) reveals the important roles of the hub genes, *JAK2* and *GADD45A*. The network also includes 104 intermediate genes (white circle). Solid line indicates direct interaction, and dotted line indicates indirect interaction

Since the eight genes were identified as candidate hallmark genes for HCM, the investigation of their interactions with known HCM disease genes would provide a deep insight into their biological roles. The interaction data were extracted from StringDB and Biogrid database [23, 24], and the PPI network was formed to summarize these links. As shown in Fig. 8, a total of 155 nodes and 463 edges relationship pairs were identified in the PPI network, including 44 known HCM disease genes, 7 candidate HCM hallmark genes and 104 intermediate genes. Four candidate HCM hallmark genes (*JAK2*, *MBP*, *CD209*, *TRAK2*) have both direct and indirect interactions with known HCM disease genes, while the other 3 candidate HCM hallmark genes (*C1R*, *GADD45A*, *METTL7B*) only have indirect interactions with known HCM disease genes. Among the eight candidate HCM hallmark genes, the most connected genes were *JAK2* and *GADD45A*, both with 35 underlying edges.

Further functional enrichment analysis showed that the intermediate genes were mostly transcription factors and protein kinases, which are involved in the regulation of multiple signaling transduction pathways, including protein kinase signaling pathway, calcium-mediated signaling pathway and intrinsic/extrinsic apoptotic signaling pathway, et al. Furthermore, intermediate genes that participate in positive regulation of cardiac muscle tissue growth and cardiac septum morphogenesis were also identified in the process.

### *JAK2* and *GADD45A*

The expression levels of *JAK2* and *GADD45A* with the well-established biomarkers of HCM (*NPPA* and *NPPB*) were explored using the Pearson’s correlation analysis. Positive relationship can be found between *JAK2* and *NPPA* (r = 0.62, p = 4.05e−06), *JAK2* and *NPPB* (r = 0.65, p = 8.88e−07), *GADD45A* and *NPPA* (r = 0.63, p = 2.18e−06), *GADD45A* and *NPPB* (r = 0.66, p = 5.70e−07).

The expression of *JAK2* were further explored in the heart tissues of HCM animal models. Significant difference was noted between MHC^403/+^ mice and wild type (WT) mice based on the dataset GSE52038 (p < 0.01) [28]. However, negative results were found in the other HCM animal models, probably due to the timing of the detection, since the samples from human were mostly collected in the end stage of HCM, whereas the samples from animal models were generally collected in the earlier disease stage of HCM.

## Discussion

In the last decades, two gene expression profiling technologies including microarray and RNA-Seq have been proved to be excellent in revealing the biomarkers and cellular pathways of human disease [29]. Previous studies on HCM transcriptomic data have focused on only the microarray datasets or the RNA-Seq datasets [30, 31]. Advances in bioinformatics and the increasing number of transcriptomic datasets have enabled a full exploration of the integrated transcriptomic data to reveal molecular mechanisms underlying HCM.

An exhaustive search from the GEO, ArrayExpress, and SRA public repository has been performed to collect HCM and control heart tissue samples from both technologies. After data integration and DEGs extraction, the general LFC value of the identified DEGs were relatively low, we assume that the mild change of gene expression may be related to the slow disease progression of most HCM cases.

During the classification process, SVM, RF and KNN technologies were implemented for the DEGs evaluation. The differences in performance among classification techniques are usual in this type of problems, and several papers comparing classification techniques for biological data can be found in the literature [32–34]. In the results above-mentioned, SVM classifier attains an optimal performance using only 8 genes. The behavior is also seen in the KNN technique, although with a lower performance. RF classifier obtained similar results when using the complete set of 48 genes but fails to design a simpler classifier with a low number of genes with optimal performance [32]. Thus, these results support the design of an optimal classifier based on SVM classifier with only eight genes.

The PPI network established between known HCM disease genes and eight HCM candidate hallmark genes contains helpful information for understanding the role of them in the development of HCM. *JAK2* and *GADD45A* were found to be hub genes in the PPI network, indicating their important roles underlying HCM. Further functional enrichment analysis also showed that some intermediate genes participate in positive regulation of cardiac muscle tissue growth and cardiac septum morphogenesis.

Janus kinase 2, encoded by *JAK2*, is a protein tyrosine kinase involved in a specific subset of cytokine receptor signaling pathways. As a member of JAK family, JAK2 is an important component in the Janus kinase/signal transducer and activator of transcription (JAK/STAT) signaling pathway. The JAK/STAT signaling triggers multiple signals involved in development, homeostasis and inflammation [35, 36]. Accumulating evidence indicated that the JAK/STAT signaling pathway played a vital role in transducing stress and growth signals in the hypertrophic heart [37, 38]. The JAK/STAT pathway also transduces signals for a wide array of cytokines and growth factors including ANGII, TNF-α, IL-1β, IL-6 and IFN-γ, all of which have been involved in cardiac hypertrophy [39–42]. Moreover, JAK2 has previously been reported to play an important role in left ventricular remodeling during pressure overload hypertrophy, and the development of hypertrophy can be blocked by pharmacological inhibition of JAK2 kinase [43]. Furthermore, one mutation V617F in *JAK2* has been identified in one patient with myeloproliferative disorder (MPD) and HCM, suggesting a potential causative role of JAK2 in the development of HCM phenotype [44]. Recent studies also showed that cardiac JAK2 was critical for maintaining normal heart function, and its ablation produced a severe pathologic phenotype composed of myocardial remodeling [45]. Taken together, it is likely that *JAK2* plays a central role in the pathogenesis of HCM. From our previous study, rare mutations in *JAK2* were identified in 9/72 (12.5%) HCM patients without mutations in known HCM disease genes (Table 2) [3]. It would be interesting to further explore the specific role of these mutations and their associations with HCM.

**Table 2:** Mutations in *JAK2* (NM_008413) identified in 72 non-sarcomeric HCM patients

Sample ID	Het/hom	AA change	SNP	MAF
H13, H53, H44, H07	het	N1108S	rs142269166	1.96e−3
H17	het	L892V	rs201551707	6.56e−4
H24, H51	het	L393V	rs2230723	7.88e−3
H60, H65	het	R1063H	rs41316003	4.37e−3

*MAF* minor allele frequency in GnomAD database

Another hub gene *GADD45A*, encoding growth arrest and DNA damage inducible alpha, is a member of *GADD45* gene family, which have been implicated in stress signaling responses to various physiological or environmental stressors, thus contributing to the maintenance of genomic stability [46]. Several previous studies have evaluated the hypothesis that two other GADD45 isoforms, including GADD45G and GADD45B, may have relevance to cardiac physiopathology [47, 48].

As one candidate hallmark gene, *METTL7B* is a member of mammalian methyltransferase-like family. The expression of *METTL7B* in HCM significantly decreased in our study. In line with our findings, one recent study showed that the expression levels of *METTL7B* in the cardiac tissue in the diabetic cardiomyopathy patient group were statistically lower than those in the healthy group [49]. However, the expression of *METTL7B* was significantly increased in iPSC-CMs compared with hESCs and fibroblasts. Despite the opposite results, all the present data support the importance of *METTL7B* in HCM, experimental data have yet to be fully investigated to determine its pathogenic relevance.

Additionally, the list of HCM-related genes between our study and previous studies of those datasets were compared and found that even though some genes appeared in the opposite direction in separated datasets [30, 50], most HCM relevant genes showed the same directions between microarray and RNA-seq datasets, including the eight candidate HCM hallmark genes.

Furthermore, due to the limited number of genes detected in the microarray datasets compared to the RNA-seq datasets, focusing on common DEGs through the intersection of datasets tend to lose some important information that RNA-seq would confer. Unfortunately, no enriched biological function and pathway based on the 48 identified HCM relevant genes can be found through GO and pathway analysis. However, we are confident with the results because they have been validated in different platforms and different patient cohorts.

Previous studies have demonstrated that distinct cellular pathways were involved in the development of HCM corresponding to different causative gene mutations [40]. However, based on the results in this study, we assumed that the eight candidate hallmark genes may act as a central role in the mutual cellular pathways underlying the HCM phenotype, which can somehow be triggered by most causative gene mutation. Further studies are needed to decipher the specific role of the candidate hallmark genes associated with HCM.

## Conclusions

Integrating transcriptomic datasets from different platforms, have greatly aid the utility of biological data and improved the interpretation of gene expression values. Our results showed that the pipeline has good performance and a high accuracy of the classifier to distinguish unknown samples. Additionally, the central role of *JAK2* and *GADD45A* in the pathogenic mechanism of HCM was highlighted. These findings will greatly contribute to extending our knowledge of the biological changes underlying HCM and providing perspective to reveal the pathology and develop therapeutic targets for HCM.

## Supplementary Information

**Additional file 1**. **Table S1**: The statistical information of the 48 DEGs obtained in microarray dataset. **Table S2**: The statistical information of the 48 DEGs obtained in RNA-Seq dataset. **Table S3**: Results of the three classifiers for both accuracy and f1-score when using different numbers of genes.

## Data Availability

The datasets used and analyzed during the current study are available from three public database, ArrayExpress (http://www.ebi.ac.uk/arrayexpress/), Gene Expression Omnibus (GEO, https://www.ncbi.nlm.nih.gov/gds/) and Sequence Read Archive (SRA, https://www.ncbi.nlm.nih.gov/sra/). Accession numbers of the datasets used are shown in Table 1. The code for processing the data in the study is available within fgshare (https://doi.org/10.6084/m9.figshare.14650536).

## References

[1] Elliott PM, Anastasakis A, Borger MA, Borggrefe M, Cecchi F, Charron P, Hagege AA, Lafont A, Limongelli G, Mahrholdt H (2014). 2014 ESC Guidelines on diagnosis and management of hypertrophic cardiomyopathy: the Task Force for the Diagnosis and Management of Hypertrophic Cardiomyopathy of the European Society of Cardiology (ESC). EUR HEART J.

[2] Ingles J, Burns C, Bagnall RD, Lam L, Yeates L, Sarina T, Puranik R, Briffa T, Atherton JJ, Driscoll T (2017). Nonfamilial hypertrophic cardiomyopathy: Prevalence, natural history, and clinical implications. Circ Cardiovasc Genet.

[3] Xu J, Li Z, Ren X, Dong M, Li J, Shi X, Zhang Y, Xie W, Sun Z, Liu X (2015). Investigation of pathogenic genes in Chinese sporadic hypertrophic cardiomyopathy patients by whole exome sequencing. Sci Rep.

[4] Casamassimi A, Federico A, Rienzo M, Esposito S, Ciccodicola A (2017). Transcriptome profiling in human diseases: new advances and perspectives. Int J Mol Sci.

[5] Castillo D, Galvez JM, Herrera LJ, Roman BS, Rojas F, Rojas I (2017). Integration of RNA-Seq data with heterogeneous microarray data for breast cancer profiling. BMC Bioinformatics.

[6] Nookaew I, Papini M, Pornputtapong N, Scalcinati G, Fagerberg L, Uhlen M, Nielsen J (2012). A comprehensive comparison of RNA-Seq-based transcriptome analysis from reads to differential gene expression and cross-comparison with microarrays: a case study in Saccharomyces cerevisiae. Nucleic Acids Res.

[7] Murugan A, Nair S, Kumar K (2019). Detection of skin cancer using SVM, random forest and kNN classifiers. J Med Syst.

[8] Wei L, Su R, Wang B, Li X, Zou Q, Gao X (2019). Integration of deep feature representations and handcrafted features to improve the prediction of N6-methyladenosine sites. Neurocomputing.

[9] Boateng EY, Otoo J, Abaye D (2020). Basic tenets of classification algorithms K-nearest-neighbor, support vector machine, random forest and neural network: a review. J Data Anal Inf Process.

[10] Ritchie ME, Phipson B, Wu D, Hu Y, Law CW, Shi W, Smyth GK (2015). limma powers differential expression analyses for RNA-sequencing and microarray studies. Nucleic Acids Res.

[11] Du P, Kibbe WA, Lin SM (2008). lumi: a pipeline for processing Illumina microarray. Bioinformatics.

[12] Dobin A, Davis CA, Schlesinger F, Drenkow J, Zaleski C, Jha S, Batut P, Chaisson M, Gingeras TR (2013). STAR: ultrafast universal RNA-seq aligner. Bioinformatics.

[13] Li H, Handsaker B, Wysoker A, Fennell T, Ruan J, Homer N, Marth G, Abecasis G, Durbin R (2009). The Sequence Alignment/Map format and SAMtools. Bioinformatics.

[14] Anders S, Pyl PT, Huber W (2015). HTSeq–a Python framework to work with high-throughput sequencing data. Bioinformatics.

[15] Tarazona S, Furio-Tari P, Turra D, Pietro AD, Nueda MJ, Ferrer A, Conesa A (2015). Data quality aware analysis of differential expression in RNA-seq with NOISeq R/Bioc package. Nucleic Acids Res.

[16] Hansen KD, Irizarry RA, Wu Z (2012). Removing technical variability in RNA-seq data using conditional quantile normalization. Biostatistics.

[17] Ding C, Peng H (2005). Minimum redundancy feature selection from microarray gene expression data. J Bioinform Comput Biol.

[18] Peng H, Long F, Ding C (2005). Feature selection based on mutual information: criteria of max-dependency, max-relevance, and min-redundancy. IEEE Trans Pattern Anal Mach Intell.

[19] Pedregosa F, Varoquaux G, Gramfort A, Michel V, Thirion B, Grisel O, Blondel M, Prettenhofer P, Weiss R, Dubourg V (2011). Scikit-learn: machine learning in python. J Mach Learn Res.

[20] Noble WS (2006). What is a support vector machine?. Nat Biotechnol.

[21] Diaz-Uriarte R, Alvarez DAS (2006). Gene selection and classification of microarray data using random forest. BMC Bioinformatics.

[22] Parry RM, Jones W, Stokes TH, Phan JH, Moffitt RA, Fang H, Shi L, Oberthuer A, Fischer M, Tong W (2010). k-Nearest neighbor models for microarray gene expression analysis and clinical outcome prediction. Pharmacogenomics J.

[23] Szklarczyk D, Morris JH, Cook H, Kuhn M, Wyder S, Simonovic M, Santos A, Doncheva NT, Roth A, Bork P (2017). The STRING database in 2017: quality-controlled protein-protein association networks, made broadly accessible. Nucleic Acids Res.

[24] Oughtred R, Stark C, Breitkreutz BJ, Rust J, Boucher L, Chang C, Kolas N, O'Donnell L, Leung G, McAdam R (2019). The BioGRID interaction database: 2019 update. Nucleic Acids Res.

[25] Shannon P, Markiel A, Ozier O, Baliga NS, Wang JT, Ramage D, Amin N, Schwikowski B, Ideker T (2003). Cytoscape: a software environment for integrated models of biomolecular interaction networks. Genome Res.

[26] Bindea G, Mlecnik B, Hackl H, Charoentong P, Tosolini M, Kirilovsky A, Fridman WH, Pages F, Trajanoski Z, Galon J (2009). ClueGO: a cytoscape plug-in to decipher functionally grouped gene ontology and pathway annotation networks. Bioinformatics.

[27] Lan F, Lee AS, Liang P, Sanchez-Freire V, Nguyen PK, Wang L, Han L, Yen M, Wang Y, Sun N (2013). Abnormal calcium handling properties underlie familial hypertrophic cardiomyopathy pathology in patient-specific induced pluripotent stem cells. Cell Stem Cell.

[28] Christodoulou DC, Wakimoto H, Onoue K, Eminaga S, Gorham JM, DePalma SR, Herman DS, Teekakirikul P, Conner DA, McKean DM (2014). 5'RNA-Seq identifies Fhl1 as a genetic modifier in cardiomyopathy. J Clin Invest.

[29] Ibrahim NE, Januzzi JL (2018). Established and emerging roles of biomarkers in heart failure. Circ Res.

[30] Liu X, Ma Y, Yin K, Li W, Chen W, Zhang Y, Zhu C, Li T, Han B, Liu X (2019). Long non-coding and coding RNA profiling using strand-specific RNA-seq in human hypertrophic cardiomyopathy. Sci Data.

[31] Li J, Wu Z, Zheng D, Sun Y, Wang S, Yan Y (2019). Bioinformatics analysis of the regulatory lncRNAmiRNAmRNA network and drug prediction in patients with hypertrophic cardiomyopathy. Mol Med Rep.

[32] Statnikov A, Wang L, Aliferis CF (2008). A comprehensive comparison of random forests and support vector machines for microarray-based cancer classification. BMC Bioinformatics.

[33] Statnikov A, Aliferis CF (2007). Are random forests better than support vector machines for microarray-based cancer classification?. AMIA Annu Symp Proc.

[34] Wu W, Xing EP, Myers C, Mian IS, Bissell MJ (2005). Evaluation of normalization methods for cDNA microarray data by k-NN classification. BMC Bioinformatics.

[35] Moresi V, Adamo S, Berghella L (2019). The JAK/STAT pathway in skeletal muscle pathophysiology. Front Physiol.

[36] O'Shea JJ, Schwartz DM, Villarino AV, Gadina M, McInnes IB, Laurence A (2015). The JAK-STAT pathway: impact on human disease and therapeutic intervention. Annu Rev Med.

[37] Fan Z, Gao Y, Huang Z, Xue F, Wu S, Yang J, Zhu L, Fu L (2018). Protective effect of hydrogen-rich saline on pressure overload-induced cardiac hypertrophyin rats: possible role of JAK-STAT signaling. BMC Cardiovasc Disord.

[38] Wagner MA, Siddiqui MA (2012). The JAK-STAT pathway in hypertrophic stress signaling and genomic stress response. JAKSTAT.

[39] Eid RA, Alkhateeb MA, El-Kott AF, Eleawa SM, Zaki M, Alaboodi SA, Salem AA, Aldera H, Alnamar NM, Alassiri M (2019). A high-fat diet rich in corn oil induces cardiac fibrosis in rats by activating JAK2/STAT3 and subsequent activation of ANG II/TGF-1beta/Smad3 pathway: the role of ROS and IL-6 trans-signaling. J Food Biochem.

[40] Nakamura M, Sadoshima J (2018). Mechanisms of physiological and pathological cardiac hypertrophy. Nat Rev Cardiol.

[41] Terrell AM, Crisostomo PR, Wairiuko GM, Wang M, Morrell ED, Meldrum DR (2006). Jak/STAT/SOCS signaling circuits and associated cytokine-mediated inflammation and hypertrophy in the heart. Shock.

[42] Schieffer B, Luchtefeld M, Braun S, Hilfiker A, Hilfiker-Kleiner D, Drexler H (2000). Role of NAD(P)H oxidase in angiotensin II-induced JAK/STAT signaling and cytokine induction. Circ Res.

[43] Beckles DL, Mascareno E, Siddiqui MA (2006). Inhibition of Jak2 phosphorylation attenuates pressure overload cardiac hypertrophy. Vascul Pharmacol.

[44] Gattenlohner S, Ertl G, Einsele H, Kircher S, Muller-Hermelink HK, Marx A (2008). Cardiac JAK2 mutation V617F in a patient with cardiomyopathy and myeloproliferative disease. Ann Intern Med.

[45] Gan XT, Rajapurohitam V, Xue J, Huang C, Bairwa S, Tang X, Chow JT, Liu MF, Chiu F, Sakamoto K (2015). Myocardial hypertrophic remodeling and impaired left ventricular function in mice with a cardiac-specific deletion of Janus Kinase 2. Am J Pathol.

[46] Liebermann DA, Hoffman B (2008). Gadd45 in stress signaling. J Mol Signal.

[47] Lucas A, Mialet-Perez J, Daviaud D, Parini A, Marber MS, Sicard P (2015). Gadd45gamma regulates cardiomyocyte death and post-myocardial infarction left ventricular remodelling. Cardiovasc Res.

[48] Wang J, Wang H, Chen J, Wang X, Sun K, Wang Y, Wang J, Yang X, Song X, Xin Y (2008). GADD45B inhibits MKK7-induced cardiac hypertrophy and the polymorphisms of GADD45B is associated with inter-ventricular septum hypertrophy. Biochem Biophys Res Commun.

[49] Li N, Wu H, Geng R, Tang Q (2018). Identification of Core Gene Biomarkers in Patients with Diabetic Cardiomyopathy. Dis Markers.

[50] Yang W, Li Y, He F, Wu H (2015). Microarray profiling of long non-coding RNA (lncRNA) associated with hypertrophic cardiomyopathy. BMC Cardiovasc Disord.

